# Wearable Fixation Device for a Magnetically Controllable Therapeutic Agent Carrier: Application to Cartilage Repair

**DOI:** 10.3390/pharmaceutics12060593

**Published:** 2020-06-26

**Authors:** Kyungmin Lee, Gwangjun Go, Ami Yoo, Byungjeon Kang, Eunpyo Choi, Jong-Oh Park, Chang-Sei Kim

**Affiliations:** 1 School of Mechanical Engineering, Chonnam National University, Gwangju 61186, Korea; kayla.kmlee@gmail.com (K.L.); gwangjun124@gmail.com (G.G.); eunpyochoi@chonnam.ac.kr (E.C.); 2 Korea Institute of Medical Microrobotics, Gwangju 61011, Korea; yooami1218@kimiro.re.kr (A.Y.); bkang@kimiro.re.kr (B.K.)

**Keywords:** wearable therapeutic agent fixation device, Halbach array, magnetically controllable stem cell carrier, micro-scaffold, stem cell spheroid, cartilage repair.

## Abstract

Recently, significant research efforts have been devoted toward the development of magnetically controllable drug delivery systems, however, drug fixation after targeting remains a challenge hindering long-term therapeutic efficacy. To overcome this issue, we present a wearable therapeutic fixation device for fixing magnetically controllable therapeutic agent carriers (MCTACs) at defect sites and its application to cartilage repair using stem cell therapeutics. The developed device comprises an array of permanent magnets based on the Halbach array principle and a wearable band capable of wrapping the target body. The design of the permanent magnet array, in terms of the number of magnets and array configuration, was determined through univariate search optimization and 3D simulation. The device was fabricated for a given rat model and yielded a strong magnetic flux density (exceeding 40 mT) in the region of interest that was capable of fixing the MCTAC at the desired defect site. Through in-vitro and in-vivo experiments, we successfully demonstrated that MCTACs, both a stem cell spheroid and a micro-scaffold for cartilage repair, could be immobilized at defect sites. This research is expected to advance precise drug delivery technology based on MCTACs, enabling subject-specific routine life therapeutics. Further studies involving the proposed wearable fixation device will be conducted considering prognostics under actual clinical settings.

## 1. Introduction

**Figure 1:**
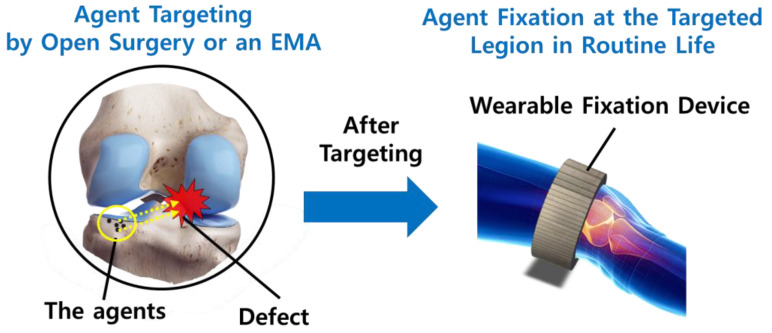
Schematic of a wearable fixation device for a magnetically controllable therapeutic agent carrier (MCTAC) with magnetic nanoparticles (MNPs) after targeting therapeutic agents onto the defect lesion.

Recently, magnetically controllable therapeutic agent carriers (MCTACs) have emerged as a suitable candidate facilitating the development of precisely targeted drug delivery systems. As MCTACs contain magnetic nanoparticles (MNPs), they can be controlled and directed to a target lesion by means of external permanent magnets or electromagnetic actuation system (EMA) [1,2,3,4]. Owing to the advantages of minimally invasive surgery and accompanied by a strong targeting ability and reduced side effects, MCTACs have been highlighted for their precise therapeutic delivery, particularly in terms of cell-based therapeutics and triggered drug releases [5]. Furthermore, most of the previous research pertaining to MCTACs has focused on the targeting mechanism and its clinical efficacy. However, the fixation of MCTACs at targeted lesions after precise delivery has not been addressed thus far. Considering the cell proliferation period, a fixation mechanism suitable for routine life after the procedure at the clinical site is essential to ensure effective therapeutics. With regard to this, we propose a novel wearable fixation mechanism and its application to an MCTAC designed for cartilage repair stem cell therapeutics, as shown in Figure 1.

Cartilage comprising chondrocytes can be damaged by external shocks, obesity, and aging, which can cause arthritis, mainly at the hip and knee joints [6]. Arthritis causes inflammation or pain due to debris released by the damaged articular cartilage. Arthritis is generally divided into four stages depending on the progression of articular cartilage damage. Conservative therapies, such as drugs and physical therapy, are employed up to stage two, where the thickness of the articular cartilage is reduced due to damage. If this damage continues, the cartilage interval in stage three further reduces to less than that in stage two. This damage is treated either with collagen or stem cells. In stage four, where the cartilage is reduced by 60%, replacement arthroplasty is performed instead of stem cell therapy. Once the cartilage is damaged, it is not easily self-repaired as the chondrocytes do not contain blood vessels [7]. Therefore, in the case of heavily damaged cartilage, therapeutics employing collagen or stem cells are used for artificial cartilage or cartilage regeneration, respectively [8,9,10,11,12,13,14,15]. In particular, research on cartilage regeneration using stem cell-based therapeutic agents has attracted significant attention. Furthermore, to achieve better performance of cell-based therapies with regard to cartilage defects, clinicians inject a polymer fleece cylinder/structure [8,9] or a large amount of secretomes of mesenchymal stem cells (MSCs) [10] into the cartilage defect area. As a majority of existing treatments based on these structures are invasive in nature, they require long recovery times and may result in scars. Moreover, the cost effectiveness of such approaches was found to be unsatisfactory owing to the significant amount of therapeutic agents that needed to be injected into the cartilage to cover defect areas.

To overcome these limitations, a novel technology involving stem cell-based therapeutic agents containing MNPs was recently proposed, based on the effects of interaction with a magnetic field. Nishijima et al. [16] used a bulk high-temperature superconducting magnet (HTS magnet) [16,17] to study the accumulation of a magnetic seeded drug in the liver of rats. An HTS magnet can generate a strong magnetic field at room temperature, however, it requires a massive cooling system and incurs high power costs. Additionally, drug delivery systems and cell-based therapies employing permanent magnets to easily generate magnetic forces have been widely researched [18,19,20]. Sawar et al. proposed a method of pushing and pulling magnetic particles into the ears of rats using two permanent magnets [21]. Barnsley et al. proposed a magnetic drug targeting system using a Halbach array, wherein, 12-in NdFeB permanent magnet elements were used [22]. However, active locomotion to the target lesion required minimally invasive surgery, which was unsuccessful due to the passive property of the permanent magnet. Therefore, to achieve external wireless active actuation, an electromagnetic system was incorporated into the MCTAC based on previous microrobot motion control technology [2,23,24]. As a promising result of the combination of the EMA and the MCTAC, Go et al. were able to develop a biodegradable scaffold with a porous structure carrying MSCs [25]. Similarly, Yoo et al. demonstrated a magnetoresponsive stem cell spheroid-based cartilage recovery platform that enabled precise targeting using magnetic control and low-frequency electromagnetic fields [13]. These methods can move and immobilize the MCTACs at the cartilage defect using a specific amount of therapeutic agents by means of an external EMA.

Although these targeting procedures have proved successful, considering the factors affecting actual clinical cell-based minimal invasive therapy, such as proliferation time, bleeds in the micro-fractured hole, and movement during daily life, it is still challenging to maintain the MCTAC at the target lesion. The MCTAC necessitates a wearable fixation device that can bridge the gap between targeting technology and actual clinical therapeutic effects. Furthermore, Go et al. demonstrated the possibility of using a single magnet to fix the targeted micro-scaffolds. However, this is a passive system that can only generate a pulling force, and the direction of the magnetic field and strength of the fixation device cannot be determined considering subject-specific conditions [25].

Therefore, to enhance the therapeutic efficacy of MCTACs, we propose a novel wearable fixation device and demonstrate its application to cartilage repair. The fixation device comprises an optimized permanent magnet array based on the Halbach magnet principle and a band that can be wrapped around the targeted body part, particularly the knees. The configuration of the Halbach magnet array is obtained via an optimization method to enable subject-specific wearable equipment that can actively interact with the MCTAC. Furthermore, we employed univariate optimization and verified the fixation performance through 2D and 3D magnetic field simulations. Finally, a prototype was fabricated, and evaluations were conducted through in-vitro and in-vivo experiments.

## 2. Materials and Methods

### 2.1. Fixation Mechanism

The proposed method is based on the magnetic interactive force between an external permanent magnet array and an MCTAC composed of MNPs. A mechanism for fixing therapeutic agents containing MNPs at the defect lesion is derived, in order to provide a magnetic field that can adequately maintain and move the MCTAC onto the target lesion. The required magnitude of the magnetic field is determined by considering the size of the MCTAC and the region of interest (ROI). Although the proposed method can be applied to an MCTAC containing agents such as stem cells [25], doxorubicin [26], macrophages [27], ferumoxytol [28], and a multifunctional nanorobot [1], this study uses stem cells as a therapeutic agent for cartilage repair in order to validate the proposed method. Here, the required magnetic field intensity is assumed to be a minimum of 40 mT, based on previous studies on magnetically actuated micro-scaffolding containing MSCs and a magnetoresponsive stem cell spheroid [12,13]. As this particular application of the proposed method is aimed at stem cell therapy through the repair of surrounding cartilage, the assumption of the desired magnetic field intensity is reasonable. Additionally, to prevent divergent movement of the MCTAC near the target site, such as the cartilage defect position in this study, the direction and focal point of the magnetic field need to be focused on the target position. Therefore, to achieve a strong and concentrated magnetic field at the desired position, we employed the Halbach array principle.

### 2.2. Fixation Device Prototype

A prototype of the wearable fixation device, comprising a band and six magnets in the configuration of a Halbach array, is fabricated for the validation of the proposed method. Each magnet in the Halbach array is customized to form a 10-mm cube of neodymium rare-earth magnet of grade N52 with a magnetization of $1.087\times 10^{6}\;A/m$. The optimal configuration of the array is obtained through univariate optimization as well as magnetic field and force simulations. Furthermore, the simulations were conducted using MATLAB (MathWorks, Natick, MA, USA), and COMSOL Multiphysics 5.0 (COMSOL, Burlington, MA, USA) to visually confirm the designed results.

### 2.3. MCTAC Type 1: Stem Cell Spheroid

For the in-vitro experiment, a spheroid [6] comprising stem cells and MNPs was used. Mouse MSCs were incubated in a culture medium containing 0.5 mg/mL MNPs (FluidMAG-D, Chemicell, Berlin, Germany) at 37 °C overnight. MCTACs containing stem cells were fabricated using Corning Ultra-Low Attachment Surface 96-well plates (Corning Costar). Dissociated mouse MSCs were resuspended in the medium (5 × 10^4^ cell/mL), and 10,000 cells/well were added to the 96-well plate and incubated at 37 °C in a humidified 5% CO_2_ atmosphere for up to 4 days.

### 2.4. MCTAC Type 2: Micro-Scaffold

For the in-vivo validation, a micro-scaffold containing MNPs was prepared. The fabrication and characterization of the micro-scaffold have been described in detail in our previous work [5]. The micro-scaffold, composed of poly-lactic-*co*-glycolic acid, was fabricated through water-in-oil-in-water (W-O-W) emulsion templating. In order to obtain the magnetic responsive property of the micro-scaffold, its surface was coated with amine-functionalized MNPs via amino bond formation.

### 2.5. Phantom Model

In-vitro experiments were conducted to confirm whether the fixation device is capable of fixing the MCTAC at the desired target. The phantom was designed by referring to the anatomy of the femur and tibia of a rabbit that was seven months old and weighed approximately 3 kg. The rabbit phantom and frame of the magnets were prepared by Veroclear RGD310 (Stratasys Ltd., Eden Prairie, MN, USA) and Verowhite photo-polymer (Stratasys Ltd., Eden Prairie, MN, USA) using a 3D printer (OBJET 30 Pro, Stratasys Ltd., Eden Prairie, MN, USA). The defect was drilled and had a diameter and depth of 2 mm. The size of the defect was determined by following the methodology provided by Duan et al. [11], and Higa et al. [29].

### 2.6. Animal Model

All the procedures involving animal subjects were performed in accordance with the ethical standards of the institutional research committee approved by Chonnam National University (CNU IACUC-YB-2019-03, 29 January 2019). In-vivo experiments using rabbits were performed to confirm that the optimized array and the simulated results would represent the results obtained using a living body. To maintain consistency with the phantom experiment, a seven-month-old male rabbit weighing approximately 3.5 kg was prepared. Moreover, the defect was punched with a diameter of 5 mm and a depth of 2 mm at the open cartilage. The experimental procedure and results were obtained using a dental surgical microscope, OPMI pico S100 (Carl Zeiss Meditec AG, Jena, Germany), and HDR-PJ675 Handycam (SONY, Tokyo, Japan). The fixation device was attached to the rabbit using a band and wrapped with a tourniquet. 

### 2.7. Magnetic Force Model

The magnitude of a magnetic field is determined by several factors, such as the grade, volume, and location of the magnet. For a given magnetization volume of the MCTAC, the generated interaction force is inversely proportional to the distance between two magnetic objects, such as the distance between the MCTAC and external magnetic source. Additionally, the magnetic field strength of a block-shaped ferromagnetic material is determined using the Biot–Savart law, as follows:

(1) $$B\left(r\right)=\frac{Br}{\pi }\left[\tan^{-1}\frac{h\;D}{2r\sqrt{4r^{2}+h^{2}+D^{2}}}-\tan^{-1}\frac{h\;D}{2\left(L+r\right)\sqrt{4{\left(L+r\right)}^{2}+h^{2}+D^{2}}}\right],$$

where B(r) is the remanent field of B, and r is the distance from the magnet to the point of interest (POI);h is the height, D is the depth, and L is the length of the magnet.

To simulate the magnetic force and direction of the designed Halbach array, we derive the magnetic force as follows. First, the magnetic force, F, of a single superparamagnetic particle is given by [22]:

(2) F=∇(μ·B)=V ∇(M·B),

where μ=M V is a moment of a single superparamagnetic particle, M is its magnetization, V is the volume of particles, and B is the magnetic flux density expressed as $B=\mu_{0}H$. The magnetization of a single superparamagnetic particle, M, driven by the Langevin function, L(y)=coth(y)−1/y, where $M_{s}=4.7\times 10^{5}A\cdot m^{-1}$ is the saturation magnetization of the superparamagnetic particle at room temperature, is given by:

(3) $$M\left(H\right)=M_{s}L\left(\frac{M_{s}V\mu_{0}H}{k_{B}T}\right),$$

where $k_{B}T$ is the result of the Boltzmann constant and temperature. The magnetic flux density of each point dipole, generated by the optimized permanent magnets array is described as:

(4) $$B_{i}\left(r^{\prime }\right)=\frac{\mu_{0}}{4\pi }\left(\frac{3r^{\prime }\left(\mu_{i}\cdot r^{\prime }\right)}{r^{\prime 5}}-\frac{\mu_{i}}{r^{\prime 3}}\right),$$

where $\mu_{0}=4\pi \times 10^{-7}H/m$ is the permeability of vacuum, $\mu_{i}=MdV$ is the point moment, and $r^{\prime }$ is the position vector of the point moment. The normalized magnetic force acting on a superparamagnetic particle can be calculated at the POI, which is the center of the ROI. Finally, the normalized magnetic force was calculated using:

(5) $$\frac{F}{M_{s}V}=\frac{M}{M_{s}}\nabla \left(B\right),$$

whose units are $T\cdot m^{-1}$. When $M=M_{s}$ is satisfied, the force is equivalent to the gradient force. In other words, the normalized magnetic force is proportional to the variance of the magnetic flux density. Using the magnetic force Equations (1)–(4) for a single magnet, the total magnetic force of the Halbach array can be computed by adding the magnetic force of each *i*-th magnet in the array:

(6) $$F_{POI}=\sum F_{i}.$$

### 2.8. Halbach Array

**Figure 2:**
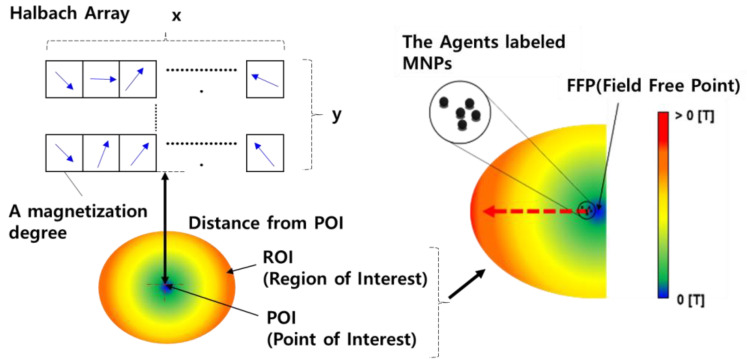
Fixation of MCTACs based on the Halbach array principle and field-free point.

Generally, when a permanent magnet and magnetic materials are in close proximity, the magnetic materials move toward the magnet; the magnetic substance moves from a low magnetic flux density position to a high magnetic flux density position, as shown in Figure 2.

Ideally, the point at which the magnetic flux density equals 0 T is called a field-free point (FFP). A concentrated magnetic field is formed at the FFP, and this field can be directed toward the desired position by changing the position of the FFP. The MCTAC can be steered toward the desired target region using this phenomenon. However, a combination of multiple magnetic fields originating from multiple permanent magnets is required to move the FFP to the desired position, indicating that a zero force position can be identified using the attraction and repulsion forces of each magnet. This is the basic concept of the Halbach array. We applied this concept to the device using multiple permanent magnets. The performance of the Halbach array has been proven in the field of industrial machinery, such as in motors [30,31,32].

The magnetization direction of the concentrated field point can be controlled by appropriately arranging two or more magnets in the Halbach array, as shown in Figure 2, where the magnetic force is increased on one side and decreased to approximately zero on the opposite side [33,34]. Thus, we can maximize the magnetic field strength at the desired position using fewer magnets, thereby achieving a lightweight wearable fixture device suitable for daily use.

### 2.9. Optimized Configuration

**Figure 3:**
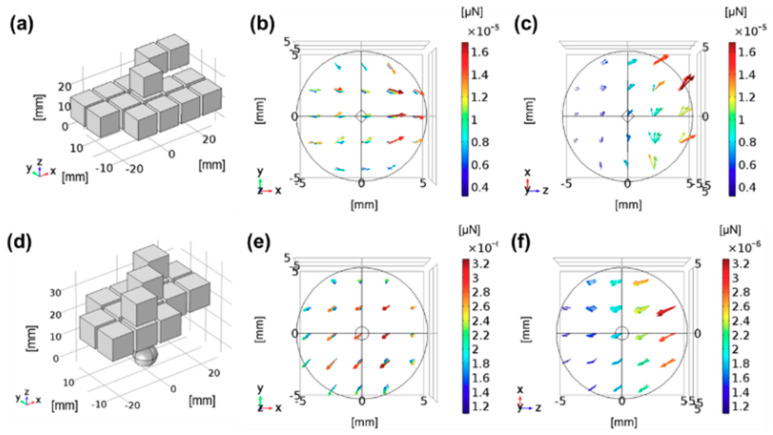
Simulation for a fixation device capable of pulling and pushing. (**a**) An exemplar optimized configuration; (**b**) the resultant magnetic field; and (**c**) the pulling force. (**d**) An exemplar configuration; (**e**) the resultant magnetic field; and (**f**) the pushing force.

The configuration of the Halbach array is determined by considering the required magnetic force and direction for the given MCTAC procedure. The parameters include the target and wearing positions; the desired direction and required magnetic force, considering the subject’s condition and size; and the number of magnets and array configuration from the perspective of device fabrication. These parameters result in redundant freedom during device design, necessitating the selection of an optimal configuration from the available sets. Figure 3 presents two example configurations for generating pushing and pulling forces, where 15 permanent magnets were used for different purposes of fixation.

In this study, we assumed that parameters related to the subjects and their anatomic condition are obtained by considering the target position, position of the wearing device, and magnetization properties of the MCTAC, in order to design a subject-specific wearable fixation device for the given procedure environment. Based on these parameters, we first decided the direction of the magnetic field in the Halbach array that can maintain the MCTAC at the target lesion. Thereafter, we applied the univariate searching algorithm to determine the best configuration of the array, with a minimum number of permanent magnets. The univariate searching method is a relatively simple, brute-force searching algorithm offering easy computation; in this algorithm, each magnetic field direction in the array was defined as a variable to minimize the number of parameters during optimization. As the direction of the placement of the permanent magnet in the array determines the required direction and force of the fixation device, we can determine the optimal configuration by comparing the resultant output from all the available permanent magnet assembly sets. The cost function to determine the optimal configuration is expressed as:

(7) $$arg\;max\;\left\{F_{POI}\right\},$$

where i is the number of magnets in the Halbach array.

The optimization method is verified through a three-dimensional Finite Element Method (FEM) simulation. In this study, the pulling direction is the direction in which the magnetic substance or the agents labeled with MNPs are facing the optimized array, whereas the pushing direction is the direction in which they move away from the optimized array when they are released at the center of the ROI.

## 3. Results and Discussions

### 3.1. Device Fabrication

**Figure 4:**
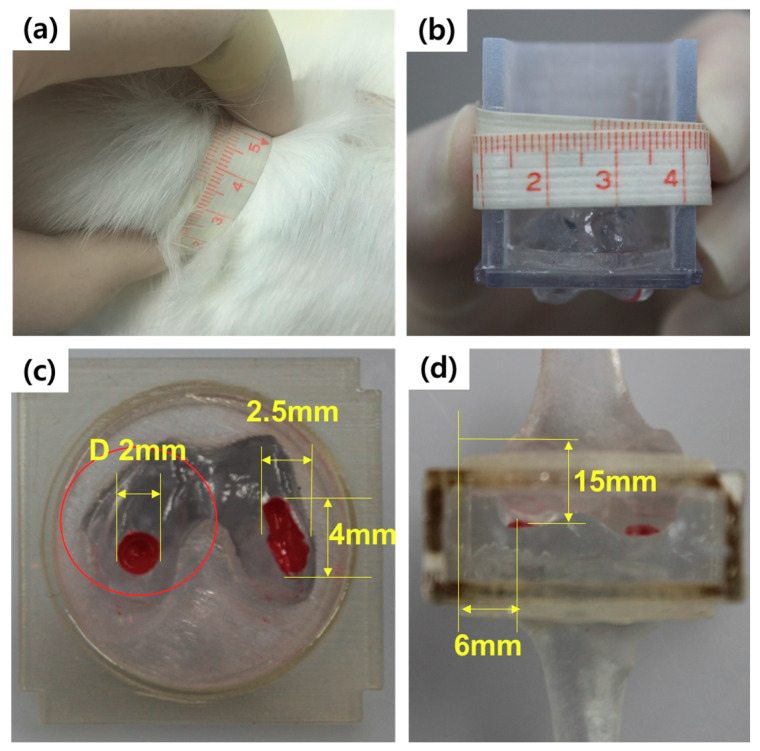
Dimensions of target animal and phantom model mimicking real environment. (**a**) Animal model, (**b**) phantom model, (**c**) top view of cartilage defect, and (**d**) side view of cartilage defect.

Figure 4 shows the actual dimensions of the animal subject knee, and the phantom model mimicking the cartilage of the subject animal. As the geometric parameters are dominant factors for the appropriate optimization and design required for the fabrication of the subject-specific wearable fixation device, we measure the actual target geometric parameters from the animal subject. As shown in Figure 4a,b, the circumference and width of the knee are measured to fabricate the 3D phantom. The artificial defect position and device wearing position are subsequently designed, as shown in Figure 4c,d. The defects are created close to the designed position on the animal cartilage, and the wearable device is wrapped over the upper part of the knee during the in-vivo experiments.

The optimization was executed based on the geometric parameters of the test subject, and the configuration of the magnetic arrays was obtained. Considering the subject’s knee and available wrapping position, the fixation device was designed to use six magnets, based on the size and volume of the permanent magnets. Although the magnetic force can be increased by increasing the number of magnets, the proposed device needs to be wearable and cover the rabbit’s knee. Thus, to realize a lightweight device, we minimized the total size of the device as a background design specification.

**Figure 5:**
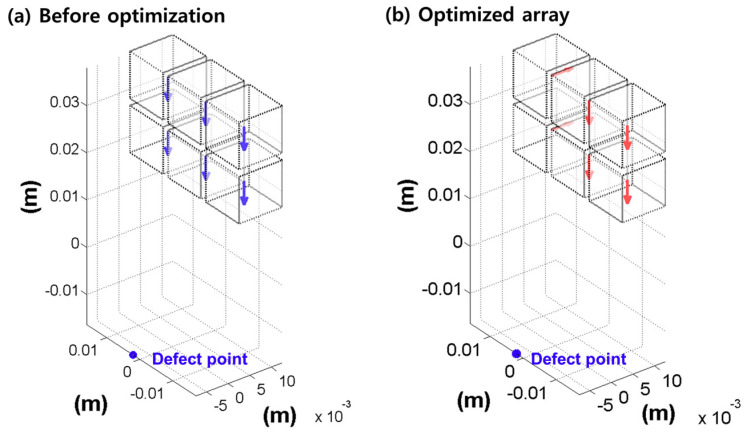
Resulting configuration of the Halbach array optimized for the given animal subject condition. (**a**) Initial configuration and (**b**) optimized configuration with different magnetic directions.

**Figure 6:**
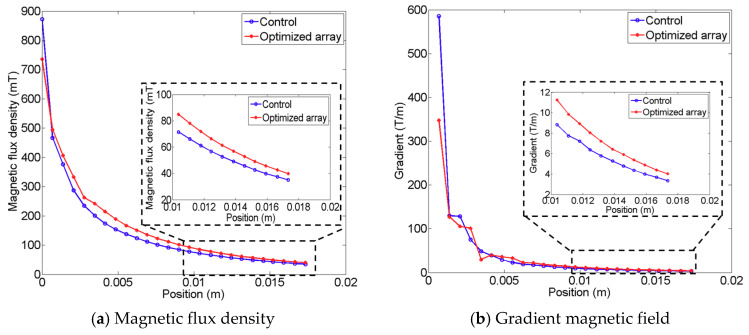
Comparison of magnetic flux density and gradient magnetic field for the initial magnetic array and the optimized magnetic array. The blue line indicates the initial configuration, and the red line represents the optimized array. (**a**) Magnetic flux density. (**b**) Gradient magnetic field.

Figure 5 shows the resultant array configuration comprising six permanent magnets, and the direction of each permanent magnet was determined to produce an enhanced pulling force at the target lesion. The figure on the left presents the initial seed configuration for the univariate optimization, and the figure on the right depicts the resultant configuration obtained through computation. In the resulting configuration, it is evident that each magnet is arranged along different directions to enhance the magnetic force at the defect lesion, while using the same six permanent magnets. The results of comparing the magnetic fields of the resultant configuration and the initial seed configuration are presented in Figure 6; the figure also indicates the enhanced magnetic flux density and gradient field. The magnetic flux density is improved by approximately 16.03%, and the gradient field is enhanced by approximately 23.61% in the ROI. Moreover, the magnetic flux density of the proposed design can produce fields exceeding 40 mT at a distance of 16 mm, which is the minimum required field strength, as reported by previous research [12,13].

**Figure 7:**
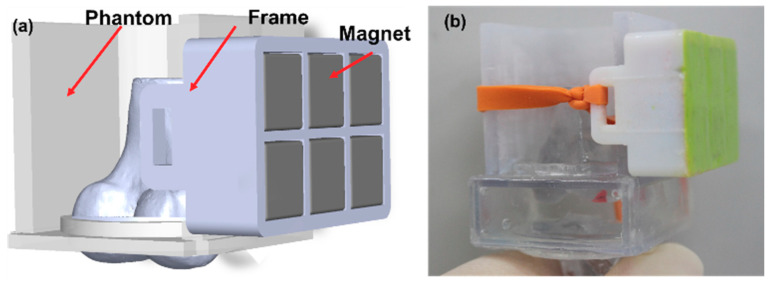
Wearable fixation device comprising a Halbach magnet array and a band wrapped around a phantom: (**a**) drawing and (**b**) prototype.

The final design and prototype of the wearable fixation device are shown in Figure 7. The device consists of the optimized array with six permanent magnets, a soft frame to assemble the magnets, and a band to attach it to the knee. The device can be wrapped over the upper part of the knee, as shown in Figure 7b.

### 3.2. Results of In-Vitro Experiment

The in-vitro experiment was conducted using the 3D phantom mimicking a 7-month-old rabbit’s cartilage, as explained in previous sections. Prior to the experiment, spheroids [6] containing MNPs were loaded into the defect lesion on the cartilage phantom model, in which the defect was created by drilling a hole with a diameter of 4 mm and depth of 2 mm, whereas the spheroid had a diameter of 300 μm. The space inside the phantom was filled with PBS solution to mimic the fluidic environment in the cartilage.

**Figure 8:**
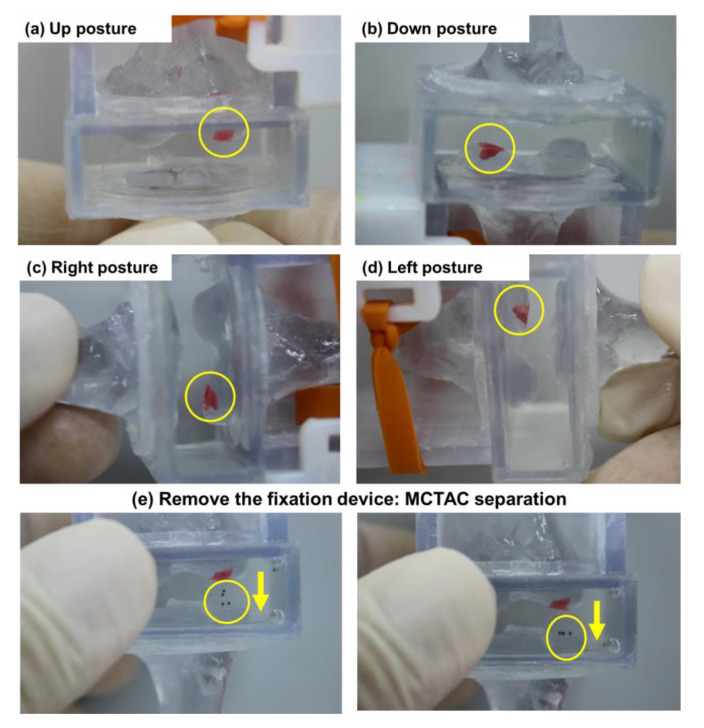
Results of the in-vitro experiments for different knee postures: (**a**) up posture, (**b**) down posture, (**c**) left posture, (**d**) right posture, and (**e**) separation of the MCTAC from the targeted lesion on removing the fixation device.

First, the fixation performance with respect to the change in knee posture was validated by varying the direction of the phantom model as shown in Figure 8a–d. 

Figure 8a–d present the results of the fixation capability with respect to different postures of the cartilage, which simulate routine changes in the posture of the knee, and the therapeutic agents were positioned at the defect marked in red. The spheroids were immobilized at the defect site when the phantom model changed its posture in any direction from 0 to 360°. Additionally, we could observe that, even with the dynamic motion and slight shaking caused by assuming extreme movements, the spheroid remained fixed at the defect by means of the fixation device. However, when the fixation device was removed from the phantom model, the targeted spheroids dropped from the targeted lesion, as shown in Figure 8e. Through these in-vitro experiments, we could confirm that the proposed fixation device was capable of fixing MCTACs at the targeted lesion in a stable manner, even in the presence of routine movements.

### 3.3. Results of In-Vivo Experiment

**Figure 9:**
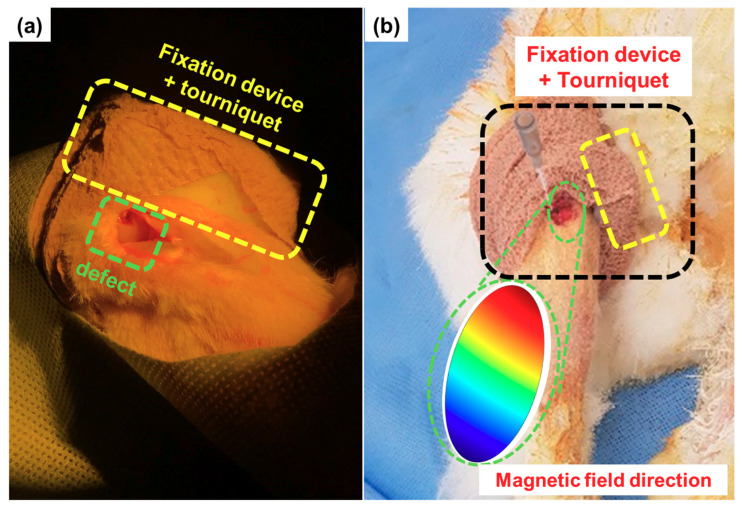
In-vivo experimental setup, where the fixation device is wrapped around the upper part of the knee of the animal subject: (**a**) fixation device secured on the rabbit using a tourniquet and (**b**) magnetic field at the defect due to the fixation device.

The in-vivo experiment was conducted to demonstrate the feasibility of the proposed method under actual anatomic environments. A male rabbit aged seven months and weighing approximately 3.5 kg was used in this animal experiment, similar to the animal subject used for the subject-specific wearable fixation device fabrication and simulation as well as the in-vitro experiments. Ten micro-scaffolds used for the stem cell delivery MCTAC, labeled with MNPs, were loaded near the defect with synovial fluid [35]. As shown in Figure 9, the fixation device was placed on the upper part of the knee, close to the designed position depicted in Figure 4. We used a tourniquet to secure the fixation device during the experiment.

**Figure 10:**
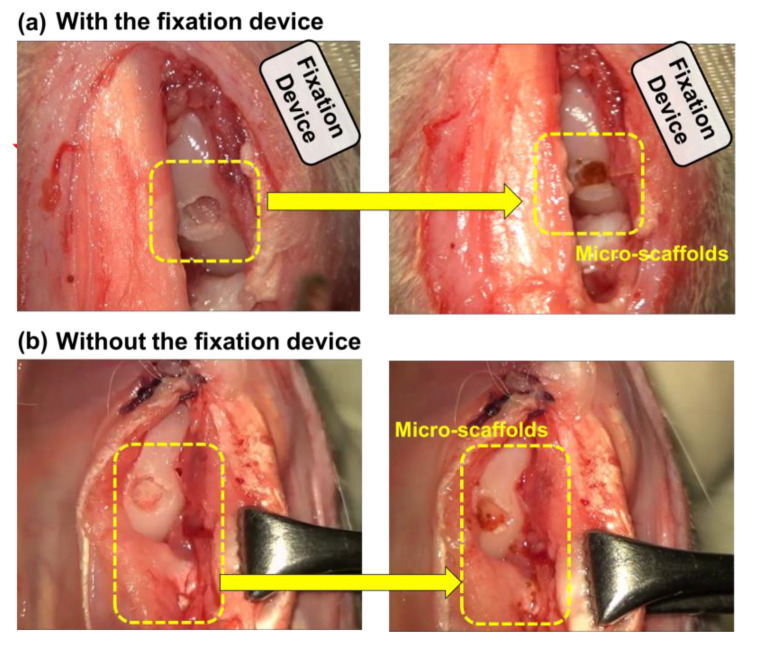
Results of the in-vivo experiment involving the injection of micro-scaffolds. (**a**) with the fixation device: the MCTAC remains fixed at the target defect, and (**b**) without the fixation device: the MCTAC flows toward the lower tissues.

Figure 10 presents the in-vivo experiments. When micro-scaffolds were injected near the defect, most of the micro-scaffolds that interacted with the fixation device were attached to the designed defect lesion (Figure 10a,b). Furthermore, the experimental results show that a majority of the micro-scaffold fell from the target area when the fixation device was not attached.

Furthermore, the strong fixation force offered by the device was also verified through several experiments. After injecting the micro-scaffold, we injected PBS solution via a disposable syringe to produce a flow similar to bleeding in the fixation area, and this flow intentionally washes out the micro-scaffolds. The results indicate that, even under this extreme condition, the micro-scaffolds remain fixed at the defect lesion by means of the fixation device. However, in the absence of the fixation device, the micro-scaffolds fail to remain attached at the defect lesion.

### 3.4. Potential Applications

Based on the experimental results and observations, we believe that the proposed subject-specific wearable fixation device for MCTACs will enhance long-term therapeutics of targeted drugs, especially for cell-based therapies, which necessitate a regeneration period. However, after fixing the MCTACs at target tissues, the MNPs used to fabricate the MCTACs remain inside the subject’s body for a significant period of time. It has been well documented that MNPs are primarily distributed in the mononuclear phagocyte system organs, such as the liver and spleen, and are relatively scarce in the lungs, heart, and kidney [36]. These MNPs bind to hemoglobin via plasma transferrin and are distributed in the Kupffer cells of the liver and removed through phagocytosis of the macrophages in the reticuloendothelial system [37]. In addition, there exist biocompatible MNPs, and the FluidMAG-D used in this study was coated with starch, which is a biocompatible compound, resulting in a toxicity lower than that of non-coated MNPs [38,39]. Thus, the proposed device features clinical applicability and shows potential for the development of precisely targeted drug delivery systems.

### 3.5. Limitations

This study demonstrates the feasibility and practical applicability of the proposed MCTAC fixation methodology through animal studies, however, several limitations and considerations for actual in-vivo application remain unaddressed. First, the adhesive force between the MCTAC, especially a micro-scaffold, and the tissue inside the body needs to be considered. In the in-vivo experiment, we ascertained that only a few micro-scaffolds were attached to the tissue near the defect and they did not move easily toward the desired position. Hence, for a minimally invasive procedure of MCTAC targeting using an EMA, the in-vivo environmental condition should be considered prior to designing the system. However, we believe that the proposed method will achieve superior performance for this application after the invasive MCTAC procedure. Second, a long-term prognosis of the proposed fixation device is required for further clinical application. As we could not account for unexpected movements of the subject, such as jumping or biting off the fixation device after the experiment, we were unable to obtain a long-term prognosis in this study. Although the in-vitro experiments yielded promising results for routine movement of a human body, it is still necessary to confirm these results for practical use. These limitations need to be overcome in the near future through additional stable experimental protocols in an in-vivo environment.

## 4. Conclusions

In summary, we proposed and validated the use of a wearable fixation device for immobilizing MCTACs. The proposed methodology was applied to design and fabricate stem cell-based therapeutic agent carriers containing MNPs for cartilage regeneration. A literature review revealed that this is the first feasible result of the fixation of MCTACs for targeted drug delivery after targeting the procedure of therapeutic agents in the defect lesion. The study strengthens the efficacy of targeted drug delivery by expanding the fixation period during routine life.

The Halbach array configuration optimized via the univariate searching method was used to generate the FFP and achieve a stronger magnetic field and force using permanent magnets of the same volume. The FFP could be generated by changing the magnetization direction of two or more permanent magnets. When the magnetic materials or the agent labeled with MNPs were released near the FFP, they moved from a region of low magnetic flux density to a region of high magnetic flux density. Moreover, the fixation device based on the Halbach array was capable of pushing and pulling the MCTAC, thus simulating the complications in fixation performances. The proposed device can be further extended to develop a targeted drug delivery system. The performance of the proposed method was verified via both 3D simulation and in-vitro and in-vivo experiments, by altering the anatomic geometric conditions of the subject animal. Through these experiments, the feasibility of the device in immobilizing MCTACs at the cartilage defect was confirmed, with regard to minor motions of the patient during routine life.

The proposed method features applicability to targeted drug delivery systems. However, several limitations related to the in-vivo environmental adhesiveness and the long-term prognostic results require further elaboration for the development of subject-specific precise drug delivery systems.
